# Reverse Logistics Network Design for Effective Management of Medical Waste in Epidemic Outbreaks: Insights from the Coronavirus Disease 2019 (COVID-19) Outbreak in Wuhan (China)

**DOI:** 10.3390/ijerph17051770

**Published:** 2020-03-09

**Authors:** Hao Yu, Xu Sun, Wei Deng Solvang, Xu Zhao

**Affiliations:** 1 Department of Industrial Engineering, UiT The Arctic University of Norway, Lodve Langesgate 2, 8514 Narvik, Norway; xu.sun@uit.no (X.S.); wei.d.solvang@uit.no (W.D.S.); 2 School of Economics and Management, China Three Gorges University, Yichang 443002, China; zhaoxu@ctgu.edu.cn

**Keywords:** epidemic outbreak, medical waste, reverse logistics, epidemic logistics, network design, operations research

## Abstract

The outbreak of an epidemic disease may pose significant treats to human beings and may further lead to a global crisis. In order to control the spread of an epidemic, the effective management of rapidly increased medical waste through establishing a temporary reverse logistics system is of vital importance. However, no research has been conducted with the focus on the design of an epidemic reverse logistics network for dealing with medical waste during epidemic outbreaks, which, if improperly treated, may accelerate disease spread and pose a significant risk for both medical staffs and patients. Therefore, this paper proposes a novel multi-objective multi-period mixed integer program for reverse logistics network design in epidemic outbreaks, which aims at determining the best locations of temporary facilities and the transportation strategies for effective management of the exponentially increased medical waste within a very short period. The application of the model is illustrated with a case study based on the outbreak of the coronavirus disease 2019 (COVID-19) in Wuhan, China. Even though the uncertainty of the future COVID-19 spread tendency is very high at the time of this research, several general policy recommendations can still be obtained based on computational experiments and quantitative analyses. Among other insights, the results suggest installing temporary incinerators may be an effective solution for managing the tremendous increase of medical waste during the COVID-19 outbreak in Wuhan, but the location selection of these temporary incinerators is of significant importance. Due to the limitation on available data and knowledge at present stage, more real-world information are needed to assess the effectiveness of the current solution.

## 1. Introduction

The number and impact of both natural and human-related catastrophes have been increasing since the 1950s [1]. Among various kinds of disasters, epidemic disease outbreaks may pose tremendous treats for human beings [2,3], and it may, if ineffectively controlled, further become a pandemic and lead to a global crisis. In accordance with the World Health Organization (WHO), an epidemic outbreak is “*the occurrence of disease cases in excess of normal expectancy*” [4], which is usually caused by an infectious disease through human-to-human transmission and animal-to-human transmission or by the exposure to radioactive and hazardous chemical sources [4]. During the latest two decades, the outbreak of several infectious and deadly diseases, i.e., the severe acute respiratory syndrome (SARS) in 2003, the Marburg hemorrhagic fever in 2007, the H1N1 influenza in 2009, the Ebola virus in 2014, and the Middle East respiratory syndrome coronavirus (MERS-Cov) in 2014, have not only caused a large number of deaths, but also severely affected the economic development of these countries. Moreover, the fear of epidemic spread has led to a global panic. 

Epidemic outbreak could usually lead to a sharp increase on the infections within a very short time, which drives a dramatically increased demand of various resources, i.e., medical staff, medical supplies, healthcare facilities, etc., in order to provide a timely and sufficient medical service, control the disease spread and minimize the economic impact. In this regard, the establishment of an effective and responsive logistics network to deal with this temporarily and drastically increased demand is of essential importance. Over the years, optimization models and methods have been formulated in order to investigate the logistics problems and to improve the decision-makings for preparation of and quick response to the outbreak of infectious diseases [5]. However, another significant challenge, which is the reverse logistics system for effective management of medical waste, has never been thoroughly investigated. The medical waste generation increases exponentially in epidemic outbreak and it may, if improperly collected or treated, accelerate disease spread and pose a significant risk on both medical staffs and patients. Due to this reason, the proper design of a temporary reverse logistics system for effective management of the dramatically increased medical waste and healthcare hazards in epidemic outbreak is of paramount significance. 

Since December 2019, several cases of atypical pneumonia caused by the coronavirus disease 2019 (COVID-19) has been reported in Wuhan, China [6,7], which has later been confirmed to be due to human-to-human transmission [8]. Since January 2020, the number of COVID-19 infections has increased significantly and a global emergency has been declared by the WHO on January 31th [9]. The total reported infections by February 25th (08:30 h Norway time) had increased to 79,331 in at least 30 countries [10], among which more than 97% are reported in mainland China [10]. The total death toll has reached to more than 2600 [10], which surpasses by three times the total number of deaths during the SARS outbreak in 2003 [11]. In order to control the rapid spread of the COVID-19, the epidemic center, Wuhan, a city with more than 10 million residents, was placed on effective lockdown on January 23th and the lockdown measures were shortly after implemented in the whole of Hubei Province [12]. The number of city lockdowns in China had increased to more than 80 by the beginning of February. Besides, tourist sites were closed across the country, public gatherings were cancelled and people was encouraged to stay at home [13]. All school activities were suspended [13]. and instead, online platforms have been used for teaching and learning. The implementation of lockdown policies has effectively restricted both inbound and outbound transportation in Hubei Province [6], and the mobility of people within the cities has been largely reduced [13]. In Wuhan, within two weeks, several temporary hospitals have been established to provide more clinical beds and medical service for the COVID-19 infection. This has led to significant logistics challenges in both forward and reverse directions. From the reverse logistics perspective, the rapidly increased amount of medical waste due to the COVID-19 outbreak needs to be collected and treated in a timely, safe and effective manner in order to minimize the virus spread and the risk to humans. Taking into account the real-world challenge faced in Wuhan, a novel multi-objective multi-period mixed integer linear program is modeled for the reverse logistics network design of medical waste management in epidemic outbreak, which aims at improving the decision-makings related to the temporary facilities and the transportation planning. Meanwhile, the model could provide quantitative analysis and managerial insights into the system performance.

The reminder of the paper is organized as follows: Section 2 presents an extensive literature review on previous modeling efforts in relevant topics. Section 3 gives problem description, mathematical model and solution approach. Section 4 presents a case study based on the COVID-19 outbreak in Wuhan in order to show the application of the proposed model and to provide general policy recommendations. Section 5 concludes the paper and gives an outlook for future research. 

## 2. Literature Review

Based on the scope of this research, an extensive literature review is given with a focus on: (1) epidemic logistics models and (2) reverse logistics models for medical waste, respectively. The literature gaps and the motivation of the study are given later in this section.

### 2.1. Epidemic Logistics Models

From a general perspective, the epidemic logistics problem belongs to emergency or disaster logistics management that focuses on four consecutive decision-makings associated with risk mitigation, preparation, response activities, and post-disaster recovery [1,14]. Liu et al. [5] provided an extensive overview of logistics challenges and models for decision-makings under epidemic outbreak. Boomee et al. [1] compared the performance of several network design models in different stages of decision-making. Taking into account six infectious diseases, Adivar and Selen [15] provided an extensive literature review on the applications of epidemic modeling and the policy-making strategy.

In recent years, modeling efforts have been predominantly given to the study of epidemic diffusion with compartmental models and to the short-term planning of temporary logistics systems in order to provide highly responsive healthcare service and medical relief efforts [16]. One of the most significant logistics challenges is to set up temporary healthcare facilities in order to deal with the rapid increase of infections. In this regard, Büyüktahtakın et al. [2] formulated an mixed integer program to minimize the overall amount of infections and fatalities considering the spatial spread of an infectious disease. The model determines the number, the capacity, and the locations of patient treatment centers as well as the patient flows. Practical insights were obtained from a case study of the outbreak of Ebola virus in three African regions.

Another important logistics challenge is to allocate medical reliefs in a timely and appropriate fashion. He and Liu [17] investigated a two-level methodological framework in order to provide timely and responsive relief distribution under public health emergencies. The first model forecasts the demand of medical reliefs in different periods and the second model determines the allocation of these reliefs. Taking into account the dynamics of infections and demands, Liu and Zhao [18] formulated an optimization model to determine the replenishment strategy and the allocation plan of emergency reliefs under bioterrorism situations. Liu and Liang [19] proposed a dynamic linear program to minimize the overall cost of a three-level emergency logistics system for the allocation of medical reliefs in disease outbreak. Considering both epidemic spread dynamics and stochastic incubation period, Wang, et al. [20] developed a bi-objective stochastic model to simultaneously improve the responsiveness and the cost effectiveness of emergency resource distribution. A genetic algorithm was applied to solve the complex optimization problem. Liu and Zhang [16] investigated a mixed integer program for an epidemic resource allocation problem considering dynamic updates of demand forecast.

### 2.2. Reverse Logistics Models for Medical Waste

Reverse logistics refers to the management of various forms of returned flows in a supply chain [21]. The focus of reverse logistics is to maximize the value recovery from end-of-life (EOL) and end-of-use (EOU) products through reuse, re-fabrication, remanufacturing, recycling, and energy recovery [22,23]. Reverse logistics network design determines the configuration and the operational strategy of a reverse logistics system, which has a significant impact on its long-term performance. Over the last two decades, quantitative optimization models and computational methods have been extensively investigated in order to improve the decision-making on reverse logistics network design at both strategic and operational levels [22,24]. With the implementation of different techniques, these decision-support models focused mainly on the optimization of economic efficiency [25,26,27,28], environmental impact [23,28,29,30], potential job creation as well as other socio-economic impact factors [31]. The effectiveness of these optimization models and computational methods have been validated in a large variety of industries and business sectors [32,33,34,35].

Early research works of reverse logistics network design for medical waste management were provided by Shi, et al. [36] and Shi [37], where mixed integer programs were applied to minimize the overall cost for setting up and operating the system. Budak and Ustundag [38] proposed a single-objective mixed integer program for making the optimal location-allocation decisions related to collection, transportation and disposal of medical waste in Turkey. The primary objective of the model is to minimize the total cost. Mantzaras and Voudrias [39] developed a nonlinear integer program for the location-routing problem to minimize the overall cost for the medical waste reverse logistics network design in a Greek region. However, the problems in these studies were mainly modeled as general reverse logistics systems. The risk and environmental impact of medical waste treatment were not taken into consideration. To tackle this problem, He et al. [40] investigated the material flow and the structure of medical waste collection systems in China in order to minimize the risk of reverse logistics operations. Thakur and Anbanandam [41] studied the barriers of medical waste reverse logistics in India with a fuzzy-matrice d’impacts croisés multiplication appliquée á un classement (MICMAC) analysis. Wang, et al. [42] proposed a bi-objective mixed integer program combined with a Grey prediction model for reverse logistics network design of medical waste, which aims at minimizing both operating cost and environmental risk. The two objective functions were combined with a weighted sum and the model was validated with a real-world case study in Shanghai, China.

### 2.3. Literature Gap and Contributions of this Research

The literature survey has revealed that the modeling efforts on epidemic logistics models have predominantly focused on the forward supply chain in order to deliver medical service and distribute medical reliefs in a responsive manner. On the other hand, the research focus of the reverse logistics models for medical waste is to provide robust strategic decisions and to suggest operational strategies for the system design in a cost effective and environmentally friendly way. However, to our knowledge, no research work has been conducted to design a temporary reverse logistics system for the treatment of rapidly increased medical waste and healthcare hazards in epidemic outbreak, which, if inappropriately managed, may pose a significant risk. In order to fill the literature gap, this paper proposes a novel reverse logistics network design model for effective management of medical waste in epidemic outbreak.

The main objectives of this research are summarized as follows:

1. First, the research aims to identify and analyze the characteristics of the temporary reverse logistics system for effective management of medical waste in epidemic outbreak.
2. Considering these characteristics, the research aims to formulate an optimization model in order to improve the decision-makings on the time and the locations for setting up temporary facilities and on the operating strategies in different periods.
3. Finally, the research aims to show the application of the proposed model through a real-world case study and to obtain general managerial implications based on the computational results.

## 3. The Model

This section presents the problem description, formulates the mathematical model and develops the solution approach.

### 3.1. Problem Description

Medical waste refers to the waste materials generated due to healthcare activities from hospitals, clinics, laboratories as well as other healthcare and research institutions [42,43]. The composition of medical waste may include infectious waste, sharp object, chemical substance, pathological waste, radioactive waste, etc. [43], which may contain highly hazardous substances and may impose potential risks to medical staffs, patients and the general public [43]. The reverse logistics of medical waste comprises of three main activities: (1) collection and separation at the sources; (2) transportation to respective facilities; and (3) proper treatment and disposal [44]. At the sources, medical waste is usually collected and sorted with color-coded containers based on their characteristics, but the color selected, the waste classification and the requirement of maximum storage time in different regions are by no means identical [45]. For example, in Hubei Province, the maximum storage time of the medical waste at hospitals from the COVID-19 patients is 24 hours, while it is 48 hours for other medical waste [46]. The transportation of medical waste usually comprises of two parts, where the first part is to transport the infectious medical waste from hospitals to treatment centers and the second part is to send the residue to landfill [47]. Due to the hazardous nature, medical waste must be properly treated before the final disposal, otherwise it may has a significant environmental impact, i.e., contamination of surface and underground waters [43]. Medical waste can be treated by several methods, i.e., thermal processes, chemical processes, irradiation technologies, biological processes and mechanical processes [48], among which the incineration of medical waste is the most widely practiced method today [43]. However, the fly ash, SO_2_ as well as other pollutants from the incineration of medical waste may result in air pollution and an improper location of incineration plant may hence impose a significant risk on nearby residents [49].

**Figure 1:**
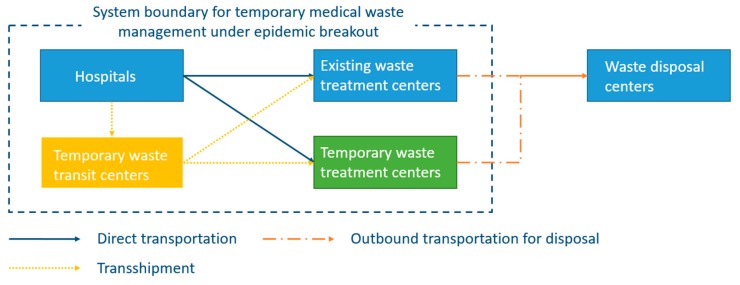
Reverse logistics network for effective management of medical waste in epidemic outbreak.

Reverse logistics network design for medical waste management has been formulated by several researches [42]. The problem is usually modeled as a two-stage decision-making problem where the first stage decisions select the strategic locations for different facilities and the second stage decisions determine the operations of the network in a short-term horizon. The focus is to balance the trade-off between economic performance and environmental risk related to collection, transportation, treatment and disposal of medical waste [42]. However, when an epidemic disease breaks out, the generation of infectious medical waste as well as the other healthcare hazards may be drastically increased within a very short period due to the exponentially rapid spread of the disease at the initial stage. This leads to a significant challenge for the reverse logistics system of medical waste and puts forward another complex short-term decision-making problem on how to deal with the fast accumulation of medical hazards. In this regard, Figure 1 presents the framework of a reverse logistics system for effective management of medical waste in epidemic outbreak, where, besides the existing facilities, temporary transit centers and temporary treatment centers are established in order to provide sufficient capacity to treat the increased medical waste and to effectively eliminate the infectious virus before disposal. The medical waste collected at hospitals as well as other healthcare centers can either be directly transported to the treatment centers or be transferred and consolidated via temporary transit centers. Nevertheless, a specific transportation mode may be implemented in accordance with practical situations.

Compared with traditional reverse logistics network design for medical waste management, the problem in epidemic outbreak has the following characteristics:

- The planning horizon is much shorter and ranges normally from several weeks to several months.
- However, within the planning horizon, the increase or change of medical waste generation is much more significant depending on the pattern of epidemic spread.
- In order to deal with the rapid change of medical waste generation, temporary facilities need to be installed in a timely and responsive way.
- Compared with the cost, the control of risk impact plays a more important role in order to effectively control the rapid spread of infectious disease.

Based on the discussion above, a multi-period multi-objective mixed integer programming model is proposed for the decision-support of reverse logistics network design for effective management of medical waste in epidemic outbreak. The objective is, through optimizing the decisions on the time and locations for setting up temporary facilities and on the transportation strategies in different periods, to responsively deal with the tremendous increase of medical waste within the planning horizon and to effectively reduce the risk of epidemic spread from the collection, transportation and treatment of medical waste and healthcare hazards. In addition, the risk from the residues of medical waste is minimized at treatment centers and can thus be safely stored, transported and disposed in later stages, so the risk associated with the transportation, storage and disposal of the residues is not taken into account in the mathematical model.

### 3.2. Mathematical Model

First, the definitions of sets, parameters and decision variables are given. Then, the objective functions and constraints of the mathematical model are formulated.

#### 3.2.1. Notations

Sets
 H, h 
Set and index of hospitals as well as other sources of medical waste
 T, t 
Set and index of the candidate locations for temporary transit centers
 E, e 
Set and index of existing treatment centers for medical waste
 D, d 
Set and index of the candidate locations for temporary treatment centers
 P, p 
Set and index of periodsParameters
${\;\mathrm{PbA}}_{h}\;$
Probability of accidental risk at hospital h∈H 

${\;\mathrm{PbA}}_{t}\;$
Probability of accidental risk at temporary transit center t∈T 

${\;\mathrm{PbA}}_{e}\;$
Probability of accidental risk at existing treatment center e∈E

${\;\mathrm{PbA}}_{d}\;$
Probability of accidental risk at temporary treatment center d∈D 

${\;\mathrm{Npat}}_{h}^{p}\;$
Number of patients received at hospital h∈H in period p∈P 

${\;\mathrm{Pop}}_{e}$
Population exposure around existing treatment center e∈E 

${\;\mathrm{Pop}}_{d}\;$
Population exposure around temporary treatment center d∈D 

${\;\mathrm{Pop}}_{\mathrm{ht}}\;$
Population exposure along the route from hospital h∈H to temporary transit center t∈T 

${\;\mathrm{Pop}}_{\mathrm{te}}\;$
Population exposure along the route from temporary transit center t∈T to existing treatment center e∈E

${\;\mathrm{Pop}}_{\mathrm{td}}\;$
Population exposure along the route from temporary transit center t∈T to temporary treatment center d∈D 

${\;\mathrm{Pop}}_{\mathrm{he}}\;$
Population exposure along the route from hospital h∈H to existing treatment center e∈E 

${\;\mathrm{Pop}}_{\mathrm{hd}}\;$
Population exposure along the route from hospital h∈H to temporary treatment center d∈D 

 RI 
Infection rate of the epidemic disease
${\;\mathrm{Gw}}_{h}^{p}\;$
Generation of medical waste at hospital h∈H in period p∈P 

${\;\mathrm{Cap}}_{t}\;$
Capacity of temporary transit center t∈T 

${\;\mathrm{Cap}}_{e}\;$
Capacity of existing treatment center e∈E 

${\;\mathrm{Cap}}_{d}\;$
Capacity of temporary treatment center d∈D 

${\;\mathrm{LB}}_{t}\;$
Lower bound utilization requirement of temporary transit center t∈T 

${\;\mathrm{LB}}_{e}\;$
Lower bound utilization requirement of existing treatment center e∈E 

${\;\mathrm{LB}}_{d}\;$
Lower bound utilization requirement of temporary treatment center d∈D 

${\;\mathrm{InS}}_{t}\;$
Installation cost of temporary transit center t∈T

${\;\mathrm{InS}}_{d}\;$
Installation cost of temporary treatment center d∈D

${\;\mathrm{Oc}}_{t}^{p}\;$
Fixed operating cost of temporary transit center t∈T in period p∈P 

${\;\mathrm{Oc}}_{e}^{p}\;$
Fixed operating cost of existing treatment center e∈E in period p∈P 

${\;\mathrm{Oc}}_{d}^{p}\;$
Fixed operating cost of temporary treatment center d∈D in period p∈P 

${\;\mathrm{pc}}_{t}^{p}\;$
Unit processing cost at temporary transit center t∈T in period p∈P 

${\;\mathrm{pc}}_{e}^{p}\;$
Unit treatment cost at existing treatment center e∈E in period p∈P 

${\;\mathrm{pc}}_{d}^{p}\;$
Unit treatment cost at temporary treatment center d∈D in period p∈P 

${\;\mathrm{tc}}_{\mathrm{ht}}^{p}\;$
Unit transportation cost from hospital h∈H to temporary transit center t∈T 

${\;\mathrm{tc}}_{\mathrm{te}}^{p}\;$
Unit transportation cost from temporary transit center t∈T to existing treatment center e∈E

${\;\mathrm{tc}}_{\mathrm{td}}^{p}\;$
Unit transportation cost from temporary transit center t∈T to temporary treatment center d∈D 

${\;\mathrm{tc}}_{\mathrm{he}}^{p}\;$
Unit transportation cost from hospital h∈H to existing treatment center e∈E 

${\;\mathrm{tc}}_{\mathrm{hd}}^{p}\;$
Unit transportation cost from hospital h∈H to temporary treatment center d∈D 
Decision variables
${\;Y}_{t}\;$
Binary variable determines if a temporary transit center will be opened at candidate location t∈T

${\;Y}_{d}\;$
Binary variable determines if a temporary treatment center will be opened at candidate location d∈D

${\;\mathrm{OT}}_{t}^{p}\;$
Binary variable determines if a temporary transit center t∈T will be used in period p∈P 

${\;\mathrm{OT}}_{e}^{p}\;$
Binary variable determines if an existing treatment center  e∈E will be used in period p∈P 

${\;\mathrm{OT}}_{d}^{p}\;$
Binary variable determines if a temporary treatment center  d∈D will be used in period p∈P 

${\;\mathrm{UQ}}_{h}^{p}\;$
Uncollected amount of medical waste at hospital h∈H in period p∈P 

${\;Q}_{t}^{p}\;$
Amount of medical waste processed at temporary transit center t∈T in period p∈P 

${\;Q}_{e}^{p}\;$
Amount of medical waste treated at existing treatment center  e∈E in period p∈P 

${\;Q}_{d}^{p}\;$
Amount of medical waste treated at temporary treatment center  d∈D in period p∈P 

${\;Q}_{\mathrm{ht}}^{p}\;$
Amount of medical waste transported from hospital h∈H to temporary transit center t∈T in period p∈P 

${\;Q}_{\mathrm{te}}^{p}\;$
Amount of medical waste transported from temporary transit center t∈T to existing treatment center e∈E in period p∈P 

${\;Q}_{\mathrm{td}}^{p}\;$
Amount of medical waste transported from temporary transit center t∈T to temporary treatment center d∈D in period p∈P 

${\;Q}_{\mathrm{he}}^{p}\;$
Amount of medical waste transported from hospital h∈H to existing treatment center e∈E in period p∈P 

${\;Q}_{\mathrm{hd}}^{p}\;$
Amount of medical waste transported from hospital h∈H to temporary treatment center d∈D in period p∈P 


#### 3.2.2. Objective Functions

The objective of the mathematical model is to balance the trade-off between overall risk and economic performance for the effective management of dramatically increased generation of medical waste and healthcare hazards in epidemic outbreak. Different from the other logistics factors, i.e., cost, lead-time, etc., risk is a relatively abstract concept and is thus difficult to be quantified accurately. Several methods have been investigated for the quantification and assessment of risk in different fields [50,51,52]. Equation (1) presents a widely accepted approach for evaluating the risk impact [53], where the risk impact is determined by the probability and the consequences of a risk event. For implementing this risk assessment approach to a variety of projects or systems, different parameters should be defined accordingly. In this regard, both quantitative methods, i.e., AHP [54,55], TOPSIS [55], etc., and qualitative methods, i.e., Delphi method [56], expert options [57], etc., have been extensively investigated for the quantification of the probability and the consequence of risk events:

(1) Risk=Probability × Consequence 

Based on Equation (1), two objective functions are formulated in order to minimize the risk at the sources of medical waste and the risk related to the transportation and treatment of medical waste. The first objective function Equation (2) minimizes the risk at hospitals, healthcare centers as well as other sources of medical waste, where large amounts of patients are received within a short period for medical service in epidemic outbreak. The probability of accidental risk at the sources is measured by the professional level and by the capacity of medical waste management at different hospitals and healthcare institutions. Research has revealed improper management of healthcare hazards results in severe consequence of HIV infections of medical staffs [58]. Furthermore, the risk of the spread of infectious disease increases rapidly if the healthcare hazards cannot be collected and treated in a timely and appropriate manner. For example, researches have shown the persistence of coronavirus on inanimate surfaces, i.e., medical waste, could be up to 9 days [59]. Moreover, some infectious diseases, i.e., the COVID-19, may be possible for aerosol transmission especially within closed environment like hospitals [60], which further increase the risk of disease spread. The consequence of accidental risk is proportional to the number of patients received at hospitals and healthcare institutions and to the spread rate of the epidemic disease. Therefore, for a given epidemic disease, the objective aims at minimizing the long-time storage of medial waste at the sources in order to reduce of the risk of disease spread among patients and medical staffs due to accidents:

(2) $$\mathrm{Minimize}\;f_{1}=\;\underset{p\in P}{\sum }\underset{h\in H}{\sum }PbA_{h}UQ_{h}^{p}Npat_{h}^{p}RI$$

The second objective function, Equation (3), minimizes the risk related to the transportation and treatment of medical waste. Due to the highly infectious nature, medical waste is hazardous and can thus be modeled based on several established hazardous location-routing problems [61,62,63,64,65]. The first three parts evaluate the transportation risk via temporary transit centers and the last two parts calculate the risk related to direct transportation. The probability of transportation risk is measured by the probability of traffic accident and by the amount of the medical waste transported in each route. The consequence of transportation risk is proportional to the size of population along the route. The sixth part calculates the processing risk at temporary transit centers and the last two parts evaluate the treatment risk at both existing and temporary treatment centers. The treatment risk is related to the probability of accident at different facilities, the amount of medical waste received, and the population exposure. It is noteworthy that the historical or statistical data of accident may not be available for temporarily established transit centers and treatment facilities. In this regard, experts’ opinions may be important to determine the model inputs:

(3) $$\begin{array}{ll} \mathrm{Minimize}\;f_{2} & =\underset{h\in H}{\sum }\underset{t\in T}{\sum }\underset{p\in P}{\sum }PbA_{ht}Q_{ht}^{p}Pop_{ht}+\underset{t\in T}{\sum }\underset{e\in E}{\sum }\underset{p\in P}{\sum }PbA_{te}Q_{te}^{p}Pop_{te}\\ & +\underset{t\in T}{\sum }\underset{d\in D}{\sum }\underset{p\in P}{\sum }PbA_{td}Q_{td}^{p}Pop_{td}+\underset{h\in H}{\sum }\underset{e\in E}{\sum }\underset{p\in P}{\sum }PbA_{he}Q_{he}^{p}Pop_{he}\\ & +\underset{h\in H}{\sum }\underset{d\in D}{\sum }\underset{p\in P}{\sum }PbA_{hd}Q_{hd}^{p}Pop_{hd}+\underset{t\in T}{\sum }\underset{p\in P}{\sum }PbA_{t}Q_{t}^{p}Pop_{t}\\ & +\underset{e\in E}{\sum }\underset{p\in P}{\sum }PbA_{e}Q_{e}^{p}Pop_{e}+\underset{d\in D}{\sum }\underset{p\in P}{\sum }PbA_{d}Q_{d}^{p}Pop_{d} \end{array}$$

Equation (4) minimizes the total cost for establishing and operating the temporary reverse logistics system for medical waste in epidemic outbreak, which comprises of the installation cost of temporary facilities, the facility operating cost, and the transportation cost. Even though the primary objective of the model is for risk control in disease outbreak, this objective may provide a rough cost estimation for reducing the risk impact to a certain level. based on which, a budgetary constraint may be set up by the decision-makers:

(4) $$\begin{array}{ll} \mathrm{Minimize}\;f_{3} & =\underset{t\in T}{\sum }Y_{t}InS_{t}+\underset{d\in D}{\sum }Y_{d}InS_{d}+\underset{t\in T}{\sum }\underset{p\in P}{\sum }\left(Oc_{t}^{p}OT_{t}^{p}+pc_{t}^{p}Q_{t}^{p}\right)\\ & +\underset{e\in E}{\sum }\underset{p\in P}{\sum }\left(Oc_{e}^{p}OT_{e}^{p}+pc_{e}^{p}Q_{e}^{p}\right)+\underset{d\in D}{\sum }\underset{p\in P}{\sum }\left(Oc_{d}^{p}OT_{d}^{p}+pc_{d}^{p}Q_{d}^{p}\right)\\ & +\underset{h\in H}{\sum }\underset{t\in T}{\sum }\underset{p\in P}{\sum }tc_{ht}^{p}Q_{ht}^{p}+\underset{t\in T}{\sum }\underset{e\in E}{\sum }\underset{p\in P}{\sum }tc_{te}^{p}Q_{te}^{p}+\underset{t\in T}{\sum }\underset{d\in D}{\sum }\underset{p\in P}{\sum }tc_{td}^{p}Q_{td}^{p}\\ & +\underset{h\in H}{\sum }\underset{e\in E}{\sum }\underset{p\in P}{\sum }tc_{he}^{p}Q_{he}^{p}+\underset{h\in H}{\sum }\underset{d\in D}{\sum }\underset{p\in P}{\sum }tc_{hd}^{p}Q_{hd}^{p} \end{array}$$

Apparently, the three objective functions are conflicting with each other. The minimization of one objective may lead to a weaker performance on the others. For instance, the minimization of the accidental risk at hospitals and healthcare institutions requires that a maximum amount of medical waste needs to be removed from the sources and be properly treated in a timely manner. However, on the other hand, this increases the risk and the cost of medical waste transportation and treatment. Hence, the focus of the optimization is to balance the trade-off among the three objective functions through decision-makings on the locations of temporary facilities and on the transportation strategies of each period within the planning horizon.

#### 3.2.3. Model constraints

The model is restricted by Equations (5)–(35).

*Flow balance constraints*: Equations (5) and (6) calculate the uncollected amount of medical waste at each hospital by the end of each period. Herein, $Gw_{h}^{p}$ is a time-varying state parameter that is affected by the developing tendency of an epidemic. Several models and methods have been developed to study and to predict the dynamics of an infectious disease, among which the Susceptible-Exposed-Infected-Recovered (SEIR) model is the most widely adopted one [14,16,18,20]. Equations (7) and (8) are the flow balance constraints at the temporary transit centers. In epidemic reverse logistics system, the transit centers are considered as cross-docking centers only for the consolidation of transportation and, due to the high risk, medical waste cannot be stored at the temporary transit centers. Equations (9) and (10) determine the input amount of medical waste at both existing and temporary treatment centers. It is noteworthy that the formulated network structure allows both direct transportation and transshipment of medical waste. However, considering practical situations, a specific transportation mode may be determined by setting the respective parts in the model to 0:

(5) $$UQ_{h}^{1}=Gw_{h}^{1}-\underset{t\in T}{\sum }Q_{ht}^{1}-\underset{e\in E}{\sum }Q_{he}^{1}-\underset{d\in D}{\sum }Q_{hd}^{1},\;\forall h\in H$$

(6) $$\;UQ_{h}^{p}=Gw_{h}^{p}+UQ_{h}^{p-1}-\underset{t\in T}{\sum }Q_{ht}^{p}-\underset{e\in E}{\sum }Q_{he}^{p}-\underset{d\in D}{\sum }Q_{hd}^{p},\;\forall h\in H,\;p\in P\cap p\neq 1\;$$

(7) $$\;\underset{h\in H}{\sum }Q_{ht}^{p}=Q_{t}^{p},\;\forall t\in T,\;p\in P\;$$

(8) $$\;Q_{t}^{p}=\underset{e\in E}{\sum }Q_{te}^{p}+\underset{d\in D}{\sum }Q_{td}^{p},\;\forall t\in T,\;p\in P\;$$

(9) $$\;Q_{e}^{p}=\underset{t\in e}{\sum }Q_{te}^{p}+\underset{h\in H}{\sum }Q_{he}^{p},\;\forall e\in E,\;p\in P\;$$

(10) $$\;Q_{d}^{p}=\underset{d\in D}{\sum }Q_{td}^{p}+\underset{h\in H}{\sum }Q_{hd}^{p},\;\forall d\in D,\;p\in P\;$$

*Capacity and utilization constraints:* Equation (11) gives the capacity constraints of temporary transit centers, which also require the medical waste cannot be sent via a transit center if it is not in operation. In order to guarantee the effective use of a temporary transit center, Equation (12) sets up a lower bound for the rate of facility utilization if it is selected to operate in a given period. Equations (13)–(16) are the capacity and utilization constraints of both existing and temporary treatment centers:

(11) $$Q_{t}^{p}\leq Cap_{t}OT_{t}^{p},\;\forall t\in T,\;p\in P$$

(12) $$\;Q_{t}^{p}\geq LB_{t}Cap_{t}OT_{t}^{p},\;\forall t\in T,\;p\in P\;$$

(13) $$\;Q_{e}^{p}\leq Cap_{e}OT_{e}^{p},\;\forall e\in E,\;p\in P\;$$

(14) $$\;Q_{e}^{p}\geq LB_{e}Cap_{e}OT_{e}^{p},\;\forall e\in E,\;p\in P\;$$

(15) $$\;Q_{d}^{p}\leq Cap_{d}OT_{d}^{p},\;\forall d\in D,\;p\in P\;$$

(16) $$\;Q_{d}^{p}\geq LB_{d}Cap_{d}OT_{d}^{p},\;\forall d\in D,\;p\in P\;$$

*Facility selection and operation periods:* Equations (17) and (18) ensure that, in any period, a temporary facility cannot be used when the respective candidate location is not selected. It is noteworthy that, in this mathematical model, the variables $Y_{t}$ and $Y_{d}$ determine if the respective temporary facilities are to be installed and the variables $OT_{t}^{p}$ and $OT_{d}^{p}$ decide when these temporary facilities are to be used. Equations (19) and (20) require that, if a candidate location is selected to install a respective temporary facility, it must be used for the transshipment or for the treatment of medical waste within the planning horizon:

(17) $$OT_{t}^{p}\leq Y_{t},\;\forall t\in T,\;p\in P$$

(18) $$\;OT_{d}^{p}\leq Y_{d},\;\forall d\in D,\;p\in P\;$$

(19) $$\;\underset{p\in P}{\sum }OT_{t}^{p}\geq Y_{t},\;\forall t\in T\;$$

(20) $$\;\underset{p\in P}{\sum }OT_{d}^{p}\geq Y_{d},\;\forall d\in D\;$$

*Requirements of decision variables:* Equations (21)–(34) give the domains of the decision variables, where the variables related to facility location and utilization are integers and the others related to allocation and transportation are continuous variables:

(21) $$Y_{t}\in \left\{0,\;1\right\},\;\forall t\in T$$

(22) $$\;Y_{d}\in \left\{0,\;1\right\},\;\forall d\in D\;$$

(23) $$\;OT_{t}^{p}\in \left\{0,\;1\right\},\;\forall t\in T,\;p\in P\;$$

(24) $$\;OT_{e}^{p}\in \left\{0,\;1\right\},\;\forall e\in E,\;p\in P\;$$

(25) $$\;OT_{d}^{p}\in \left\{0,\;1\right\},\;\forall d\in D,\;p\in P\;$$

(26) $$\;UQ_{h}^{p}\geq 0,\;\forall h\in H,\;p\in P\;$$

(27) $$\;Q_{t}^{p}\geq 0,\;\forall t\in T,\;p\in P\;$$

(28) $$\;Q_{e}^{p}\geq 0,\;\forall e\in E,\;p\in P\;$$

(29) $$\;Q_{d}^{p}\geq 0,\;\forall d\in D,\;p\in P\;$$

(30) $$\;Q_{ht}^{p}\geq 0,\;\forall h\in H,\;t\in T,\;p\in P\;$$

(31) $$\;Q_{te}^{p}\geq 0,\;\forall t\in T,\;e\in E,\;p\in P\;$$

(32) $$\;Q_{td}^{p}\geq 0,\;\forall t\in T,\;d\in D,\;p\in P\;$$

(33) $$\;Q_{he}^{p}\geq 0,\;\forall h\in H,\;e\in E,\;p\in P\;$$

(34) $$\;Q_{hd}^{p}\geq 0,\;\forall h\in H,\;d\in D,\;p\in P\;$$

### 3.3. Solution Approach

The three objective functions in the proposed mathematical model evaluate both risk and cost related to collection, transportation, and treatment of medical waste in epidemic outbreak. However, due to the measurements of these three objective functions are by no means identical, they cannot be combined directly with a weighted sum. Thus, in order to solve the multi-objective optimization problem, an interactive fuzzy approach proposed by Pishvaee and Razmi [66] is used in this paper.

The procedures of the interactive fuzzy approach are given as follows:

(1) First, the priority level of the three objective functions $f_{1}\;$, $f_{2}$ and $f_{3}$ are determined by the decision-makers.
(2) The best solution $f^{optimal}$ and the worst solution $f^{nadia}$ of the three objective functions are calculated. The $f^{optimal}$ is obtained by optimizing each objective function individually. The $f^{nadia}$ is calculated with a lexicographic method based on the given priority level of the objective functions in order to obtain non-dominated efficient solutions [67]. The range of each objective function can then be determined.
(3) The satisfaction level of each objective function can be calculated by the fuzzy membership functions given in Equations (35)–(37), which ranges from 0 to 1.
  (35) $$\mu_{1}\left(x\right)=\{\begin{array}{c} 1,\;\mathrm{if}\;f_{1}<f_{1}^{optimal}\;\\ \frac{f_{1}^{nadir}-f_{1}}{f_{1}^{nadia}-f_{1}^{optimal}},\;\mathrm{if}\;f_{1}^{optimal}\leq f_{1}\leq f_{1}^{nadia}\\ 0,\;\mathrm{if}\;f_{1}>f_{1}^{nadir} \end{array}$$
  (36) $$\;\mu_{2}\left(x\right)=\{\begin{array}{c} 1,\;\mathrm{if}\;f_{2}<f_{2}^{optimal}\;\\ \frac{f_{2}^{nadir}-f_{2}}{f_{2}^{nadia}-f_{2}^{optimal}},\;\mathrm{if}\;f_{2}^{optimal}\leq f_{2}\leq f_{2}^{nadia}\\ 0,\;\mathrm{if}\;f_{2}>f_{2}^{nadir} \end{array}\;$$
  (37) $$\;\mu_{3}\left(x\right)=\{\begin{array}{c} 1,\;\mathrm{if}\;f_{3}<f_{3}^{optimal}\;\\ \frac{f_{3}^{nadir}-f_{3}}{f_{3}^{nadia}-f_{3}^{optimal}},\;\mathrm{if}\;f_{3}^{optimal}\leq f_{3}\leq f_{3}^{nadia}\\ 0,\;\mathrm{if}\;f_{3}>f_{3}^{nadir} \end{array}\;$$
(4) With the given priority level of different objective functions, the multi-objective optimization problem can then be converted to a single objective model with the ε-constraint method given in Equation (38), where $\varepsilon_{f2}$ and $\varepsilon_{f3}$ are the required satisfaction levels:
  (38) $$\mathrm{Maximize}\;\mu_{1}\left(x\right)\;\mathrm{Subject}\;\mathrm{to}\text{:}\;\mu_{2}\left(x\right)\geq \varepsilon_{f2}\;\mu_{3}\left(x\right)\geq \varepsilon_{f3}\;\varepsilon_{f2},\;\varepsilon_{f3}\in \left[0,\;1\right]\;\mathrm{Equations}\;(5)–(34)$$
(5) The satisfaction levels $\varepsilon_{f2}$ and $\varepsilon_{f3}$ can be adjusted in order to generate a set of Pareto optimal solutions, from which a preferred combination can be selected by the decision-makers.

## 4. Case Study

In order to show the application of the proposed mathematical model, a case study is presented in this section based on the outbreak of a novel coronavirus in China. Besides, general managerial implications are discussed from the analysis of the computational results.

### 4.1. Data Generation

Since December 2019, a novel coronavirus has spread rapidly across China and has resulted in a significant increase of infections within a very short period. The virus was first reported in Wuhan, which is the capital of Hubei Province and it has the highest number of infections [6]. At the time of the case study, the outbreak of the COVID-19 was still at the developing stage and the available knowledge and information were thus extremely limited. Based on the information published by the Health Commission of Hubei Province (web: wjw.hubei.gov.cn) and the Wuhan Municipal Health Commission (web: wjw.wuhan.gov.cn) until February 9th (19:30 h Norway time), the COVID-19 spread tendency in Wuhan was predicted with the SEIR method in AnyLogic simulation package. The SEIR method is based on a compartment theory and has been extensively used to predict epidemic spread [68]. The SEIR method comprises of consecutive processes connecting four fundamental population groups: the susceptible population, the exposed population, the infected population, and the recovered population [69]. The dynamics of the conversion of these four types of population is introduced by Liu et al. [14] and Liu and Zhang [16].

**Figure 2:**
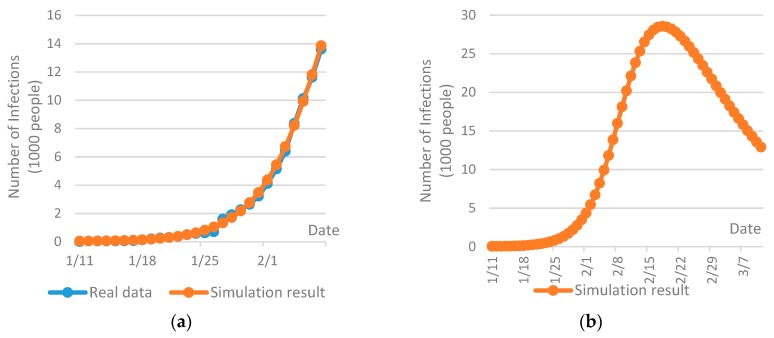
Prediction of the tendency of the COVID-19 spread in Wuhan with SEIR method in AnyLogic simulation: (**a**) Curve fit with the real data; (**b**) Predication of the infections in two months.

The AnyLogic simulation package is a powerful tool for the analysis of system dynamics [70], which has been extensively used for analysis and prediction of epidemic spread [71,72]. In this research, a SEIR model was built up in AnyLogic. The model input was, to our best, the information collected at the time of the case study (February 9th). In addition, several assumptions were made in order to predict the future disease spread tendency in Wuhan. The initial infections were set to 41, which was the reported number until January 11th [7]. The basic reproduction rate of the COVID-19, which depicts the average number of secondary infections caused by one patient to all susceptible population throughout the whole course of its infection [14], was set to 2.68 in Wuhan [6]. The incubation period of the COVID-19 was set to 7 days [73]. The average length of stay in hospital was set to two to three weeks [74] and the probability of contact infection was assumed to be 0.4. Taking into account the fact that Wuhan has been placed on effective lockdown since January 23th, 2020, the inbound and outbound transportation has been effectively cut off and the mobility of people within the city has been drastically restricted, so the disease may only be spread within a flexible domain of susceptible population. Due to this reason, the size of susceptible population was estimated with the best fit of the real data at the time of the case study, as shown in Figure 2a. The repetition of the simulation was set to 20 times and the confidence level was set to be within 10%.

Figure 2b gives a prediction of the COVID-19 spread tendency in Wuhan from January 11th to March 11th, 2020, which indicates a significant increase of infections is expected at the end of February. Then, the number of infections may be reduced continuously from the beginning of March. Based on the prediction, the planning of six consecutive periods was performed with each period includes 10 days.

By February 8th, 28 hospitals had been opened for the patients infected by the COVID-19 and two temporary hospitals have been established at Huoshenshan and Leishenshan [75]. Besides, another 11 temporary mobile cabin hospitals have been opened or planned at large public facilities, i.e., exhibition centers, sport stadiums and public schools, etc., for the treatment of mildly infections [76]. The names and capacities of these hospitals, temporary hospitals and temporary mobile cabin hospitals are given in Appendix A Table A1 and Table A2. The construction of temporary hospitals and temporary mobile cabin hospitals were finished at the beginning of February, so the patients could not be allocated to them in the first two periods within the planning horizon. For the 27 hospitals opened for the COVID-19 infections, their existing capacities are estimated by the number of opened beds and their full capacities are estimated by the number of total beds. In order to allocate the patients to different hospitals, the following rules are assumed in this paper.

(1) In the first two periods, if the total existing capacity is not exceeded, patients are proportionally distributed to the existing 28 hospitals based on their existing capacities. Otherwise, patients are proportionally allocated based on their full capacities.
(2) In the other periods, if the total existing capacity is not exceeded, patients are proportionally allocated to all the 40 hospitals based on the existing capacities. Otherwise, patients are proportionally distributed based on the full capacities of these hospitals.
(3) If the full capacity is exceeded, the full capacity of all the hospitals are utilized. In this case, the patients who cannot get a hospital bed are assumed to be under home quarantine.

The amount of medical waste and healthcare hazards generated at different hospitals is proportional to the number of infections received and the average waste generation per bed. An early study reveals that the daily generation of medical waste in China is 0.68 kg/bed [77]. In Wuhan, based on the total number of hospital beds, the average utilization rate and the total amount of healthcare waste generation given in the Wuhan Statistical Yearbook 2018 [78], the daily generation of medical waste is approximately 0.6 kg/bed. However, the treatment of infectious diseases requires much more medical resources [79], i.e., medical masks, protective glasses, and protective clothing, etc., so it leads to a higher rate of medical waste generation. Due to this reason, we assumed the daily generation rate of medical waste of the COVID-19 patients is 2.5 kg/bed. Then, the medical waste generation at different hospitals in each period was calculated by $2.5\times Npat_{h}^{p}$ kg.

**Figure 3:**
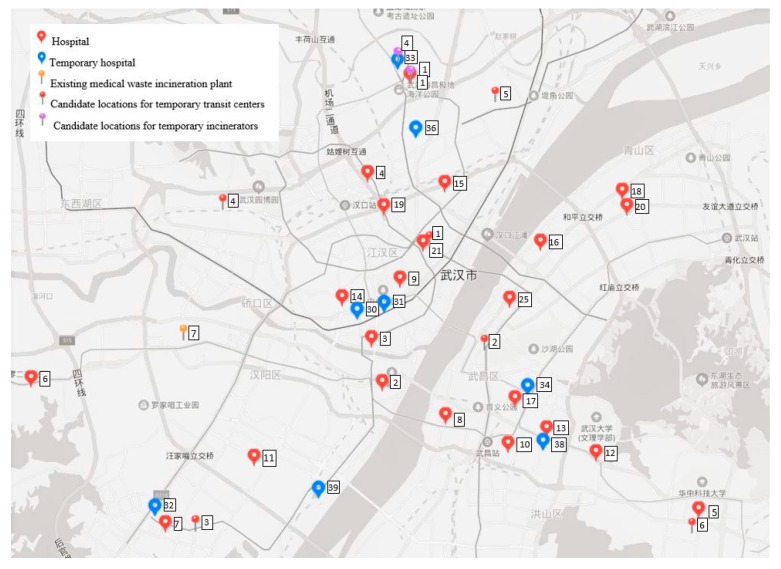
Locations of hospitals, existing facilities, and candidates for temporary facilities in central Wuhan.

The medical waste in Wuhan is currently treated at a specialized facility located at the Guodingshan incineration plant, which has a capacity of approximately 18,000 ton/year [80]. Apart from the ordinary medical waste, it is assumed 60% capacity of this plant can be used to deal with the highly infectious waste from the COVID-19 patients. Due to the sharp increase of medical waste generation, temporary incineration facilities have been planned and installed at several temporary healthcare centers, e.g., Jinyintan hospital, Huoshenshan hospital and Leishenshan hospital [81]. In addition, considering the fairness to all the hospitals based on geographical distribution, we assumed another three temporary medical waste incinerators might be opened next to three temporary mobile cabin hospitals located at Wuhan Keting, China Optics Valley Convention & Exhibition Centre and Huangpi No. 1 middle school. Thus, in total, six candidate locations for establishing temporary treatment centers were selected. In order to better consolidate the transportation of medical waste, we assumed six existing transfer stations for municipal solid waste could be converted to processing medical waste with proper technological updates, so they were assumed the candidate locations for medical waste transit centers. The lists of the candidate locations for temporary facilities are given in Appendix A Table A3 and Table A4. With the help of Baidu Maps (https://map.baidu.com/), the locations of respective nodes could be given and the distance between two nodes could be calculated. Figure 3 shows the locations of hospitals, existing facilities, and candidate locations for temporary facilities in the city center of Wuhan.

In accordance with the level of hospitals and healthcare centers, based on expert opinions, we assumed three different probabilities of accidental risk at the sources: 0.003 for the third-level Grade-A hospitals, 0.004 for the other hospitals of the COVID-19 infections, and 0.007 for the temporary hospitals and the temporary mobile cabin hospitals, respectively. The basic reproduction rate of the COVID-19 was used for the value of RI [6]. The probability of transportation risk is determined by the probability of traffic accident and is proportional to the transport distance. For infectious healthcare hazards, it can be quantified by $PbA_{ht}=0.36\times \mathrm{travel}\;\mathrm{distance}\;(\mathrm{km})\times 10^{-6}$ [65]. The consequence was assumed to be proportional to the population exposure within 500 m bandwidth along the transportation route [82], which was estimated by the travel distance and the demographic distribution of Wuhan. To evaluate the risk related to facility operations, in accordance with Zhao and Huang [65], the probability of accident was assumed to be 0.0001 for transit stations and 0.0006 treatment centers. The population exposure to medical waste transit and treatment facilities was considered as the main parameter for measuring the consequence of risk event. Herein, the facility risk was estimated by $PbA=\pi r^{2}{(\mathrm{km}}^{2})\times {\mathrm{Population}\;\mathrm{density}\;(\mathrm{people}/\mathrm{km}}^{2})$ [83], where the affected radius was set to 1 km for transit stations and 3 km for treatment centers.

Table 1, Table 2 and Table 3 present the relevant cost parameters and capacities of both existing and temporary facilities. The size and cost parameters for setting up the same-type temporary facilities at different locations were set to identical value and were assumed based on Wang, Huang and He [42], Zhao and Huang [65] and Zhao et al. [83]. The transportation cost is proportional to the travel distance. In this case study, the unit transportation cost was set to 35 yuan/ton/km [83]. Then, the transportation cost of infectious medical waste on each link was calculated. It is noteworthy that the installation cost of temporary facilities is a fixed and non-recurring cost, which only applies at the beginning of the planning based on the facility location decisions. However, the other types of cost are variable and depend thus on the usage of facilities or transportation links.

### 4.2. Result and Discussion

The optimization problems were coded and solved in Lingo 18.0 optimization solver. First, the priority levels of the objective functions were given, based on which the optimal value, the nadir value and the range of each objective function were calculated in Table 4. For analysis purpose, we set up the satisfaction levels of the risk of treatment and transportation and the total cost to 0.5 and 0.3, respectively. Then, the multi-objective optimization problem was converted to a single objective optimization and was solved. Table 5 provides the computational information for solving the optimization problem. The objective values and the satisfaction levels are given in Table 6.

In the optimal solution, the medical waste collected at hospitals is directly sent to treatment centers and the transshipment via intermediate transit centers is not selected. In addition to the existing Guodingshang medical waste incineration plant, another five candidate locations are chosen to establish temporary medical waste incinerators. Table 7 shows the facility location decisions as well as the facility usage in different periods. Furthermore, the allocation of hospitals and the facility utilization rate are given in Table 8 and Table 9, respectively. Compared with the current plan in Wuhan, both Huoshenshan and Leishenshan hospitals are selected to open temporary treatment centers. However, instead of Jinyingtan hospital, another two candidate locations at Wuhan Keting and Huangpi No.1 Middle School are chosen to install the temporary incinerators. Besides, the optimal solution suggests the operations of the temporary medical waste incinerator at Leishenshan hospital should be started from the second period, while the operations of the other incinerators should be started from the third period. Due to the time requirement on the construction, installation and adjustment of these temporary incinerators, the advanced planning of their usage is thus of significant importance.

For the existing Guodingshang medical waste incineration plant, we assumed it would be used in all periods within the planning horizon. However, as shown in Table 8 and Table 9, the utilization rate of Guodingshang medical waste incineration plant is extremely low compared with the other temporary incinerators, which means the medical waste generated at hospitals is primary distributed to temporary incinerators instead of the existing facility. The reason of this could be explained by its location. Guodingshang medical waste incineration plant is located at Hanyang district, which is an urban district and is with high population density [78]. Therefore, as argued in a previous research [49], the operations of a medical waste incineration plant not only have negative environmental impact but also pose a significant risk on the nearby residents. The medical waste and healthcare hazards generated in epidemic outbreak are highly infectious and dangerous, the treatment of them at a centrally located incineration plant may dramatically increase the treatment risk on population exposure and is thus minimized in the optimal solution.

For comparison purpose, we considered another four scenarios with different combinations of the satisfaction levels $\mu_{2}\left(x\right)$ and $\mu_{3}\left(x\right)$, as shown in Table 10. When $\mu_{2}\left(x\right)$ increases from 0.5 to 0.7, the satisfaction level of the risk at sources is reduced by 6.6% and the treatment rate in the periods 3 to 6 decreases accordingly. On the other hand, when $\mu_{2}\left(x\right)$ decreases from 0.5 to 0.3, the optimal value of $\mu_{1}\left(x\right)$ is increased from 0.91 to 0.93 and more medical waste at the hospitals are collected and treated. Taking into account the change of satisfaction level of the cost objective, the satisfaction level $\mu_{1}\left(x\right)$ is decreased by 8.8% when $\mu_{3}\left(x\right)$ increases from 0.3 to 0.4. This reveals that, for a given satisfaction level $\mu_{2}\left(x\right)\;$, a reduction on budget may lead to a higher risk at hospitals and other sources of medical waste. On the other hand, as illustrated in Table 10, an increased investment may not lead to a significant reduction on the risk level of medical waste management.

**Figure 4:**
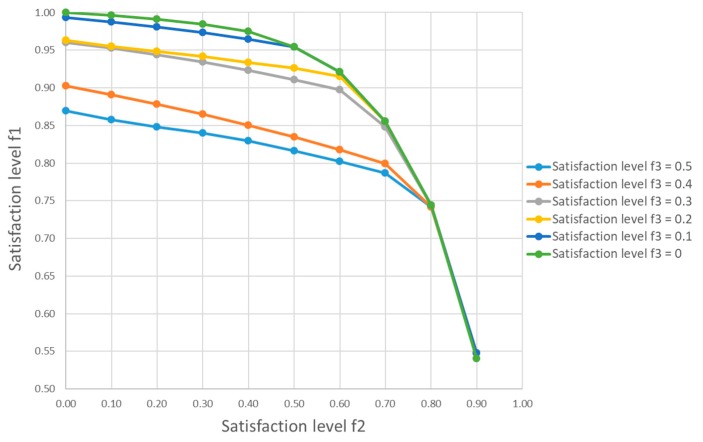
Pareto optimal solutions.

Figure 4 gives a set of efficient Pareto optimal solutions with respect to the change of the satisfaction levels $\mu_{2}\left(x\right)$ from 0 to 0.9 and $\mu_{3}\left(x\right)$ from 0 to 0.5, respectively, which clearly shows the trade-off among these three objective functions. In general, the minimization of the risk at sources may require a compromise on the risk related to transportation and treatment of medical waste. Besides, the increase of budgetary limitation for medical waste management in epidemic outbreak may result in a better performance in risk control. However, the cost effectiveness may vary drastically in different situations. For instance, in this case study, the change of $\mu_{3}\left(x\right)$ from 0.4 to 0.3 is much more effective in the risk minimization than that of decreasing $\mu_{3}\left(x\right)$ from 0.3 to 0.2.

### 4.3. Policy Recommendations and Future Discussion

Even though the case study was conduced based on several assumptions and the computational result could be affected by the input from decision-makers, four policy recommendations can still be given considering the generality of the problem:

1. The establishment of an effective epidemic reverse logistics network with temporary facilities is of significance in dealing with the rapid increase of medical waste in epidemic outbreak.
2. The selection of facility locations is one of the most important decisions for both risk control and cost management of the temporary reverse logistics system.
3. Considering the time requirement for building, installation and adjustment of temporary facilities, advanced planning of the time of facility operation is of vital importance.
4. The increase of budgetary limitation may result in a better risk control, but the cost effectiveness may vary.

**Figure 5:**
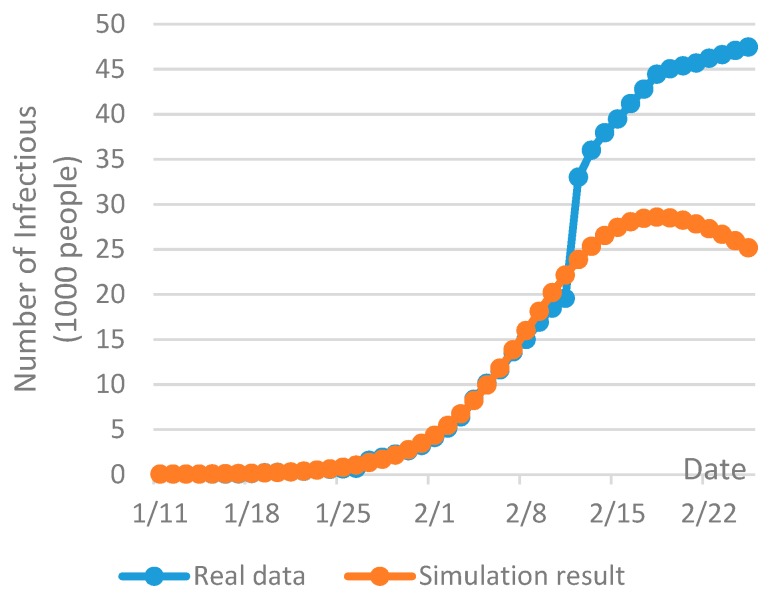
Comparison between the prediction on February 9th and the real data of the COVID-19 infections in Wuhan until February 26th.

The outbreak of the COVID-19 is at the developing stage and the future disease spread tendency is still unclear at the time of this research and is with a high level of uncertainty. As shown in Figure 5, the current infections of the COVID-19 on February 26th (08:30 h Norway time) in Wuhan have largely exceeded the prediction given by the SEIR model two weeks ago. The knowledge and information of the novel coronavirus are limited especially at the early phase of the outbreak in December 2019 [84], so the reported infections at that time may be largely variated from the real infections. In addition, due to the lack of healthcare resources and the shortage of clinical beds before the completion of those temporary hospitals in early February, a large amount of suspected infections in Wuhan cannot be diagnosed and be received in hospital in a timely way [85]. In order to provide enough clinical beds for the increased amount of the COVID-19 infections, more hospitals have been opened and more temporary mobile cabin hospitals have been planned [86]. Meanwhile, this will lead to an increase on medical waste generation and hence the capacity for medical waste management needs to be increased accordingly.

China has suffered significantly from the outbreak of the novel coronavirus. The large mobility of people may further complicate the disease control and increase future uncertainty. In addition, it is also noteworthy that the number of the COVID-19 infections reported outside of China has been sharply increased [10]. Research has revealed the risks of the COVID-19 outbreak in major cities around the globe [6]. On March 3rd (11:30 h Norway time), the total number of new infections was reported at 1804, among which 1598 new infections were reported outside China [87]. During the last one week, the rapid and significant increase on the new infections in the Republic of Korea, Italy, Japan and Iran has caused global concerns [88].

The WHO’s risk assessment on the COVID-19 outbreak at both regional and global levels have been adjusted to the highest class [87]. The keys to stop disease spread are the share of information and knowledge [84], openness and transparency [89], based on which early-stage preventive actions can be performed and rapid emergency response can be planned. As recently addressed by the Director-General of the WHO, “*with early, aggressive measures, countries can stop transmission and save lives* [88]”. Therefore, the infrastructural and resource preparedness should be done in order to effectively control a possible global outbreak of the COVID-19. Among other measures, the WHO has put focuses on providing logistics and supply chain supports for rapid response and effective control of the COVID-19 spread in many countries and areas [87]. In this regard, the model proposed in this paper may be used, not only in Wuhan but also in other major cities exposed to the risk of the COVID-19 outbreak, for the decision-support of epidemic reverse logistics network design for effective management of increased medical waste generation.

## 5. Conclusions

This paper investigates the reverse logistics network design for effective management of medical waste in epidemic outbreak, which focuses predominantly on the short-term decision-makings for the establishment and operations of a temporary system. In order to improve the location decisions of temporary facilities and the operational planning of the temporary system, a novel multi-objective multi-period mixed integer programming model is proposed in this paper. The model aims at balancing the trade-off among the risk at sources, the risk of transportation and treatment of medical waste, and the total cost. An interactive fuzzy approach was used to solve the multi-objective optimization problem and to generate a set of efficient Pareto optimal solutions. The application of the mathematical model and solution method is illustrated with a real-world case study based on the COVID-19 outbreak in Wuhan. In order to estimate the medical waste generation within the planning horizon, a SEIR model was first built up in AnyLogic simulation package. The optimization model was then solved by Lingo 18.0 based on the real data by the time of this research and several assumptions. Taking into account the generality of the problem under investigation, four policy recommendations were given for a better decision-making of the reverse logistics design for medical waste management in epidemic outbreak. In addition, the COVID-19 outbreak is at developing stage and the future spread tendency is still unclear. Particularly, it is noted the number of infections has significantly increased not only in China but also around the globe [10]. Due to this, the planning and establishment of temporary reverse logistics system for medical waste management may shortly become to a challenge in many countries. Therefore, the main contributions of this research can be summarized as follows:

(1) A novel multi-objective multi-period model is developed to optimize on the most important decisions, namely, facility location, time of operation, and transportation planning.
(2) The model focuses on the short-term decisions and the characteristics of the reverse logistics network design for medical waste management in epidemic outbreak.
(3) The applicability of the model is illustrated with a real-world case study, based on which general policy recommendations are given.

Future research may focus on the better control of high level of uncertainty with advanced methods, i.e., stochastic programming [90], robust optimization, agent-based simulation [91], etc., in order to generate robust decisions of the reverse logistics network design for medical waste management in epidemic outbreak. Furthermore, the development of more advanced and reliable methods for the prediction of the epidemic spread tendency is also of interest. In addition, the effectiveness of the current solution in Wuhan should be validated with more real-world information.

## Figures and Tables

**Table 1:** Parameters of temporary transit centers.

Installation Cost (€)	Fixed Operating Cost (€/period)	Processing Cost (€/ton)	Capacity (ton/period)
65,000	13,000	195	150

**Table 2:** Parameters of existing treatment centers.

Fixed Operating Cost (€/period)	Processing Cost (€/ton)	Capacity (ton/period)
39,000	156	300

**Table 3:** Parameters of temporary treatment centers.

Installation Cost (€)	Fixed Operating Cost (€/period)	Processing Cost (€/ton)	Capacity (ton/period)
520,000	26,000	260	100

**Table 4:** The optimal value, the worst value and the range of the objective functions.

Objective	Priority	$f^{optimal}$	$f^{nadia}$	Range
${\;f}_{1}\;$	1	0	42,336	42,336
${\;f}_{2}\;$	2	526	15,200	14,674
${\;f}_{3}\;$	3	251,361	4,615,888	4,364,527

**Table 5:** Computational information.

Optimization Solver	Value
Total variables	3701
Integer variables	84
Constraints	4301
Solver type	B-and-B
Extended solver steps	19
Total solver iterations	30,246
Runtime (seconds)	10

**Table 6:** The objective values and the satisfaction levels.

Objective	Objective Value	Satisfaction Level
${\;f}_{1}\;$	3776	0.91
${\;f}_{2}\;$	7863	0.5
${\;f}_{3}\;$	3,306,528	0.3

**Table 7:** Location decisions and facility usage in each period.

Number	Candidate Locations	Location Selection	Usage					
			p = 1	p = 2	p = 3	p = 4	p = 5	p = 6
1	Wuhan Jinyintan Hospital	0						
2	Wuhan Huoshenshan Hospital	1	0	0	1	1	1	1
3	Wuhan Leishenshan Hospital	1	0	1	1	1	1	1
4	Wuhan Keting	1	0	0	1	1	1	1
5	Optical Valley Science and Technology Exhibition Center	0						
6	Huangpi No.1 Middle School	1	0	0	1	1	1	1
7	Guodingshan Medical Waste Incineration Plant	Existing	1	1	1	1	1	1

**Table 8:** Allocation of medical waste from different hospitals in each period.

Treatment Center	Allocation of Hospitals					
	p = 1	p = 2	p = 3	p = 4	p = 5	p = 6
2			2,7,8,14,18,28,30,32,39	7,13,28,32,39	7,13,28,31,32.39	7,9,18,28,31,32,39
3		1–27	13,23,29,34,35,37,38	13,29,35,37,38	13,29,35,37,38	13,16,25,29,34,35,37,38,39
4			3,4,9,15,16,21,25,30,31,34	4,13,21,31	4,9,15,21,25,31,34	4,9,15,21,33,36
6			1,16,20,22,33,36,40	16,25,33,36	15,16,33,36	22,33,40
7	1–27			13,34		

**Table 9:** Facility utilization rate in each period.

Treatment Center	Capacity Utilization Rate					
	p = 1	p = 2	p = 3	p = 4	p = 5	p = 6
2			100%	100%	100%	100%
3		69%	100%	100%	100%	100%
4			100%	100%	100%	50%
6			100%	100%	100%	57%
7	2%			12%		

**Table 10:** Comparison of the objective value and treatment rate of medical waste at the sources in each period.

Scenarios	Objective Value	Treatment Rate at the Sources					
		p = 1	p = 2	p = 3	p = 4	p = 5	p = 6
$\mu_{2}=0.5,\mu_{3}=0.3$	0.91	100%	100%	79.1%	41.9%	39.4%	36.4%
$\mu_{2}=0.7,\mu_{3}=0.3$	0.85	100%	100%	73.7%	36.6%	23.7%	12.8%
$\mu_{2}=0.3,\mu_{3}=0.3$	0.93	100%	100%	79.1%	53.2%	42.6%	38.7%
$\mu_{2}=0.5,\mu_{3}=0.4$	0.83	100%	100%	53.6%	30.3%	23.6%	27.1%
$\mu_{2}=0.5,\mu_{3}=0.2$	0.93	100%	100%	79.1%	56.1%	43.5%	15.0%

## Appendix A

**Table A1:** Names, total beds, and number of beds opened at existing hospitals for the COVID-19 infections.

Number	Hospital	Total Beds	Number of Bed Opened
1	Wuhan Jinyintan Hospital	900	720
2	The Fifth Hospital of Wuhan	600	420
3	The Fifth Hospital of Wuhan (western hospital)	910	319
4	The Central Hospital of Wuhan (Houhu branch)	1500	515
5	The Third Hospital of Wuhan (Guanggu branch)	570	300
6	Wuhan Tongji Hospital	110	536
7	Whan Union Hospital West Campus	1200	360
8	Hubei General Hospital (Eastern Hospital)	800	420
9	Hubei Provincial Hospital of Intefrated Chinese &Western Medicine	1035	187
10	Tianyou Hospital affiliated to Wuhan University of Science & Technology	565	265
11	Wuhan Traditional Chinese Medicine Hospital (Hanyang branch)	500	100
12	Hubei Six Seven Two Integrated Traditional Chinese and Western Medicine Orthopaedics Hospital	500	273
13	General Hospital of the Central People’s Liberation Army	1800	282
14	Wuhan Lung Branch Hospital	499	122
15	Wuhan Hankou Hospital	808	304
16	Wuhan Wuchang Hospital	889	504
17	The Seventh Hospital of Wuhan	305	220
18	The Ninth Hospital of Wuhan	600	405
19	Hospital of Wuhan Red Cross Society	300	304
20	The second Hospital of WISCO	600	102
21	The Sixth Hospital of Wuhan	1200	430
22	Huangpi Hospital of Traditional Chinese Medicine	500	394
23	Jiangxia Hospital of Traditional Chinese Medicine	400	260
24	Wuhan Xinzhou District Traditional Chinese Medicine Hospital	300	200
25	Wuhan Zijing Hospital	1000	288
26	Hannan Hospital of Traditional Chinese Medicine	220	26
27	Caidian Maternity and Child Health Care Hospital	350	242
	Total beds	18,961	8498

Data source: Wuhan Municipal Health Commission [75].

**Table A2:** Names and total beds of temporary hospitals and temporary mobile cabin hospitals.

Number	Temporary Hospital and Temporary Mobile Cabin Hospital	Total Beds
28	Wuhan Huoshenshan Hospital	1000
29	Wuhan Leishenshan Hospital	1500
30	Wuhan Gymnasium (Tongkou District)	305
31	Wuhan International Conference & Exhibition Center	1800
32	Wuhan Economic & Technological Development Zone Wuhan Sports Center	1100
33	Wuhan Keting	2000
34	Hongshan Stadium	800
35	Dahuashan Outdoor Sports Center in Jiangxia District	1000
36	Wuhan National Fitness Center in Jiang’an District	1000
37	Optical Valley Science and Technology Exhibition Center, East Lake High-tech Zone	1000
38	Wuhan Shipailing Occupation Senior High School	800
39	Wuhan International Expo Center in Hanyang District	1000
40	Huangpi Gymnasium of Hubei Huangpi No.1 Middle School	300
	Total beds	13,605

Data source: Wuhan Municipal Health Commission [76].

**Table A3:** Candidate locations for temporary transit centers.

Number	Name	District	Population Density (People/km^2^)
1	Labor Waste Transfer Station	Jiang’an	11,988
2	Wuchang City Management Juxinsheng Road Garbage Transfer Station	Wuchang	19,793
3	Science Park Waste Transfer Station	Caidian	667
4	Changfeng Garbage Transfer Station	Qiaokou	21,679
5	Baibuting Garbage Transfer Station	Jiang‘an	11,988
6	Halecheng Garbage Transfer Station	Hongshan	2851

Data source of population density: Wuhan Statistical Yearbook 2018 [78].

**Table A4:** Candidate locations for temporary treatment centers.

Number	Name	District	Population Density (people/km^2^)
1	Wuhan Jinyintan Hospital	Dongxihu	1133
2	Wuhan Huoshenshan Hospital	Caidian	667
3	Wuhan Leishenshan Hospital	Jiangxia	453
4	Wuhan Keting	Dongxihu	1133
5	Optical Valley Science and Technology Exhibition Center, East Lake High-tech Zone	Hongshan	2851
6	Huangpi Gymnasium of Hubei Huangpi No.1 Middle School	Huangpi	437
7	Guodingshan Medical Waste Incineration Plant	Hanyang	5898

Data source of population density: Wuhan Statistical Yearbook 2018 [78].
